# Supplementary material for: The Construction and Exploration of a Comprehensive MicroRNA Centered Regulatory Network in Foxtail Millet (Setaria italica L.)
Source: Front Plant Sci. 2022 May 6;13:848474. doi: 10.3389/fpls.2022.848474 (PMC9121102; doi:10.3389/fpls.2022.848474)
Supplement: Supplementary file 1 [file Data_Sheet_1.zip › Sit-miRN3001b_Seita.6G230300.1_1403_TPlot.pdf]

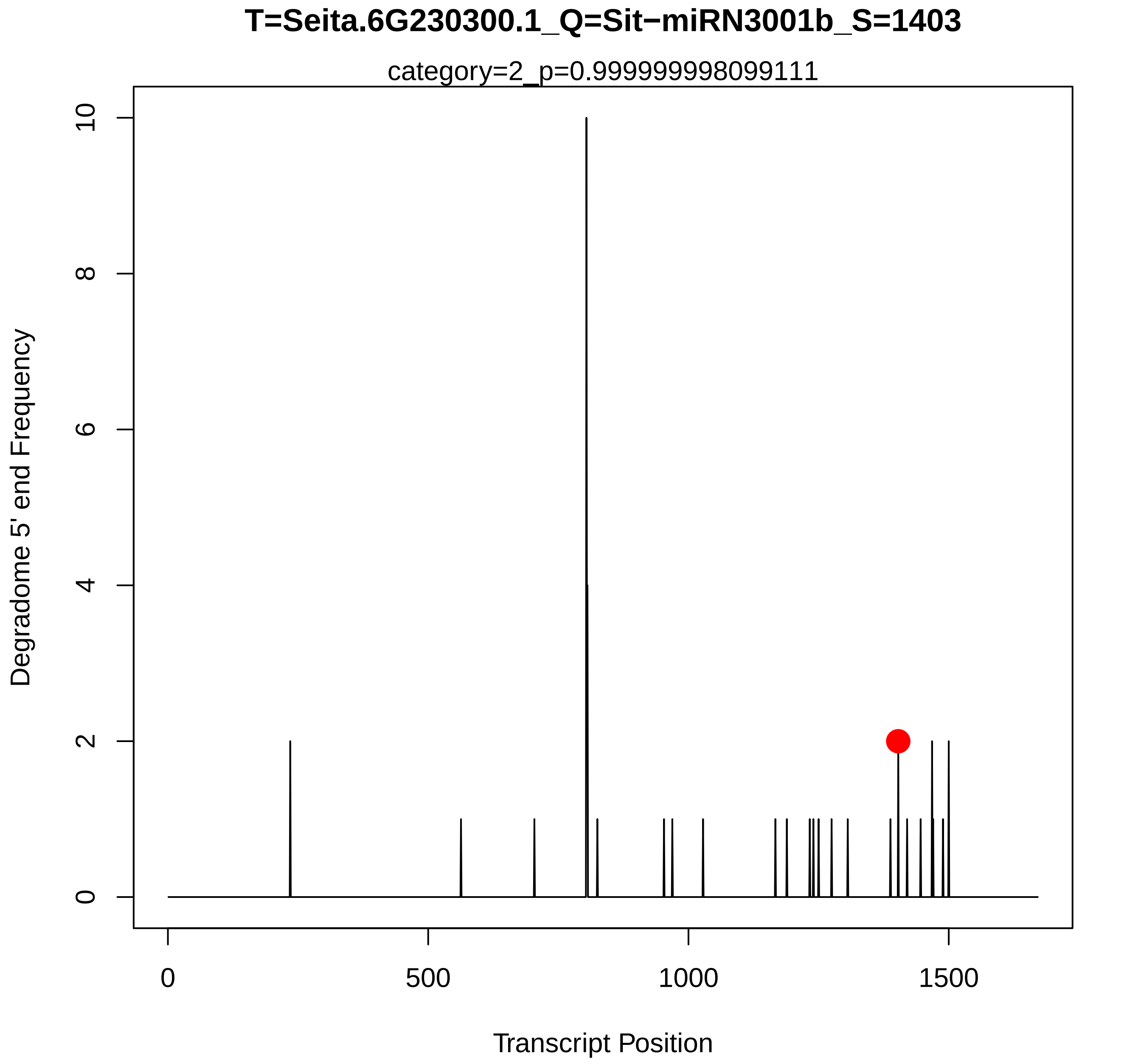

T=Seita.6G230300.1_Q=Sit−miRN3001b_S=1403
category=2_p=0.999999998099111
Degradome 5' end Frequency
0
2
4
6
8
10
0
500
1000
1500
Transcript Position
